# Development, Characterization, and Stability of Margarine Containing Oleogels Based on Olive Oil, Coconut Oil, Starch, and Beeswax

**DOI:** 10.3390/gels11070513

**Published:** 2025-07-02

**Authors:** Bárbara Viana Barbosa Naves, Thais Lomonaco Teodoro da Silva, Cleiton Antônio Nunes, Felipe Furtini Haddad, Sabrina Carvalho Bastos

**Affiliations:** 1 Nutrition Department, Federal University of Lavras, Lavars 37203-202, Minas Gerais, Brazil; vianaba9@gmail.com; 2 Food Science Department, Federal University of Lavras, Lavars 37203-202, Minas Gerais, Brazil; thaissilva@ufla.br (T.L.T.d.S.); cleiton.nunes@ufla.br (C.A.N.); felipe.haddad@ufla.br (F.F.H.)

**Keywords:** trans-fatty acid, beeswax, saturated fatty acid, lipid oxidation, organogel

## Abstract

The removal of partially hydrogenated fats, as well as the substitution of saturated fats with healthier alternatives, has become increasingly common due to their well-established association with adverse health effects. As a result, the demand for alternative formulations in the food industry has driven the development of a promising emerging technology: oleogels. Oleogels are a semi-solid material made by trapping liquid oil within a three-dimensional network formed by structuring agents. Within this context, this study aimed to develop and characterize margarines prepared with oleogels formulated from extra virgin olive oil, coconut oil, starch, and beeswax at varying concentrations. The proposed oleogel-based formulations exhibited a high melting temperature range and lower enthalpy. Although lipid oxidation levels differed between the commercial and oleogel-based margarines, they remained within acceptable limits. A significant difference in color was observed, with the oleogel formulations imparting a slight greenish hue compared to the commercial margarine. In terms of microstructure, the commercial margarine presented smaller and more uniformly distributed water droplets. Oleogel-based margarines demonstrated technological feasibility. Considering consumers’ growing interest in food innovation and health-conscious products, olive oil-based oleogel margarines represent a promising alternative, particularly due to the nutritional benefits associated with olive oil.

## 1. Introduction

The human diet plays a key role in the prevalence of chronic noncommunicable diseases, such as cardiovascular diseases. Both the quantity and the quality of the food consumed, especially fats, are extremely important in health [1]. Therefore, the elimination of partially hydrogenated fats is a key goal in the food industry due to their well-supported association with increased cardiovascular risk, and several guidelines recommend their exclusion from the diet [2].

Hydrogenated and interesterified fats are widely used in food products because they confer desirable sensory and technological properties, including consistency, specific melting characteristics, flavor, cost reduction, and extended shelf life [1]. However, the removal of these components—often rich in saturated and trans-fatty acids—presents a significant challenge for the processed food sector. As such, there is a need for fat substitutes that are technologically viable, offer sensory acceptance, maintain stability during and after processing, and meet texture and spreadability requirements [3].

In recent years, significant advances have been made in the study of oleogels, which can be used to replace trans and saturated fats in processed foods such as pastes, bars, margarines, cakes, and ice creams [4]. Oleogels are semi-solid systems formed by entrapping liquid oils within a three-dimensional network created by structuring agents [5,6].

The preparation of oleogels requires careful selection of both oils and structuring agents, with their proportions tailored to the specific food application. A successful formulation must combine components in a way that enhances functional performance. Nevertheless, challenges remain, particularly in achieving and maintaining the desired physical properties of the final product [7].

Olive oil, known for its well-documented health benefits, is widely used as a base oil in oleogel formation [8,9,10]. It is rich in monounsaturated fatty acids and is associated with numerous health benefits, including reduced risk of cardiovascular disease and cancer, lower body mass index with long-term moderate consumption, and anti-inflammatory properties [11].

Coconut oil, although predominantly composed of saturated medium-chain fatty acids, has an intermediary melting point and is extensively used in the food industry due to its structural and stabilizing properties at room temperature [12,13]. Its medium-chain triglycerides (MCTs) yield different metabolic effects compared to long-chain saturated fats, with some studies reporting increased energy expenditure and fat oxidation. Despite its high saturated fat content, moderate coconut oil consumption has been linked to favorable lipid profiles, such as increased HDL cholesterol without a significant rise in LDL cholesterol [14,15]. However, few studies have explored the combination of olive oil and coconut oil in oleogel systems, particularly in health-oriented food formulations.

Margarine is a fat-based product used both for direct consumption and as an ingredient in baking, confectionery, and homemade preparations [16,17]. While previous studies on Brazilian margarines report low trans-fat levels, they often contain high amounts of saturated fats [18]. Reducing saturated fat content improves the nutritional profile by increasing unsaturated fatty acid levels and lowering atherogenic and thrombogenic indices, thereby reducing cardiovascular risk [18]. According to the 2015–2020 Dietary Guidelines for Americans, replacing trans and saturated fats with polyunsaturated fats significantly lowers blood cholesterol and cardiovascular risk [19].

Similarly, the 2017 Brazilian Guidelines for Dyslipidemia and Atherosclerosis Prevention [20] recommend eliminating trans fats and partially replacing saturated fats with mono- and polyunsaturated fats. These recommendations are echoed in the 2019 European guidelines for dyslipidemia management [21], and the World Health Organization advises limiting trans-fat intake to less than 1% of total energy intake, while encouraging replacement of saturated fats with polyunsaturated fats [22].

Given consumers’ growing interest in healthier foods and the innovative potential of oleogel systems, margarine based on olive oil oleogels represents a promising alternative. However, a consistent challenge with wax-based oleogels is their tendency to produce an undesirable waxy mouthfeel [16,23,24]. Moreover, achieving both low saturation and zero trans fats remains difficult when reformulating water-in-oil emulsions such as margarine [18]. In this context, the present study aimed to develop a novel margarine using a multi-component oleogel with extra virgin olive oil, coconut oil, starch, and beeswax.

## 2. Results and Discussion

### 2.1. Effect of Oleogelators on Margarine Formation and Physical Properties

To optimize the texture of the margarines, the values of firmness, adhesiveness, and consistency of oleogel-based margarines were targeted to match those of a commercial margarine [25]. These individual responses were transformed into desirability values, which consolidate multiple textural attributes into a single score ranging from 0 to 1. A desirability value closer to one indicates a texture more closely resembling that of the commercial reference. Table 1 presents the experimental design matrix used to evaluate the effects of the oleogel components on texture desirability.

The concentrations of corn starch (CS), beeswax (BW), extra virgin olive oil (OO), and coconut oil (CO) were modeled using a quadratic regression to evaluate their effects on the desirability associated with the texture of a commercial margarine. The quality of the model fit was assessed using pure error analysis. The regression coefficients and results of the analysis of variance (ANOVA) are presented in Table 2 and Table 3, respectively. Among the model terms, only the interaction between CS and BW was not statistically significant (*p* > 0.05), indicating that this interaction did not have a meaningful effect on texture. All other main effects and interactions were statistically significant (*p* < 0.05), demonstrating that variations in these components had a direct influence on the textural attributes of the margarine, either enhancing or diminishing overall desirability.

According to Table 3, although the model exhibited a significant lack of fit, it still showed a satisfactory coefficient of determination (R^2^ = 0.76), and the regression was statistically significant (*p* < 0.05). This indicates that the model reasonably described the relationship between the independent and dependent variables. Furthermore, the small difference between R^2^ and the adjusted R^2^ (0.08) reinforces the adequacy of the model fit.

In general, an increase in corn starch concentration was associated with a reduction in the overall desirability, indicating a negative effect on the target texture parameters. Beeswax (BW) acted as the primary oleogelator, contributing significantly to the structure and texture of the oleogel-based margarine. This trend is consistent with previous findings, such as those reported for oleogels structured with potato starch and candelilla wax, where higher starch levels led to reduced stability, and in the absence of wax, self-sustaining gels could not be formed [26,27].

Among the formulations tested, those that achieved high desirability values (>0.8)—notably formulations 2, 4, and 9—had lower concentrations of corn starch, while formulation 12 attained a similar desirability through a markedly high BW content. These results align with other studies using oleogel systems based on wax and monoglycerides, where the mechanical properties of the gels were predominantly influenced by wax concentration rather than by other components [28,29]. However, for oleogel emulsions, the partial substitution of wax with starch appears to promote the formation of a softer and more uniform oleogel system capable of absorbing up to 45 times more water. This results in a gel with a solid-like appearance, reduced candelilla wax content, and improved spreadability [27]. These findings support the complementary structuring roles of corn starch and wax—where corn starch more effectively structures the aqueous phase and wax structures the lipid phase—with beeswax being particularly efficient due to the higher oil content in the formulation.

Based on the results described above, a numerical optimization was conducted to determine the proportions of corn starch, beeswax, extra virgin olive oil, and coconut oil that would yield a desirability value of one in the response surface model. This target desirability reflects textural attributes (firmness, adhesiveness, and consistency) closest to those of commercial margarine. As a result, three optimized margarine formulations were generated for further analysis:

Margarine formulation 1 (M1): 1.8% CS, 4.0% BW, and 73.9% combined OO and CO, with a ratio of 60% OO to 40% CO.

Margarine formulation 2 (M2): 1.8% CS, 5.2% BW, and 72.7% combined OO and CO, with a ratio of 57.5% OO to 42.5% CO.

Margarine formulation 3 (M3): 1.0% CS, 5.2% BW, and 73.5% combined OO and CO, with a ratio of 45% OO to 55% CO.

In addition to the optimized formulations prepared for further analyses, a commercial margarine sample was also evaluated to serve as a reference standard, enabling comparison of the parameters of the newly developed products with those of an existing market option.

### 2.2. Melting Behavior

**Figure 1:**
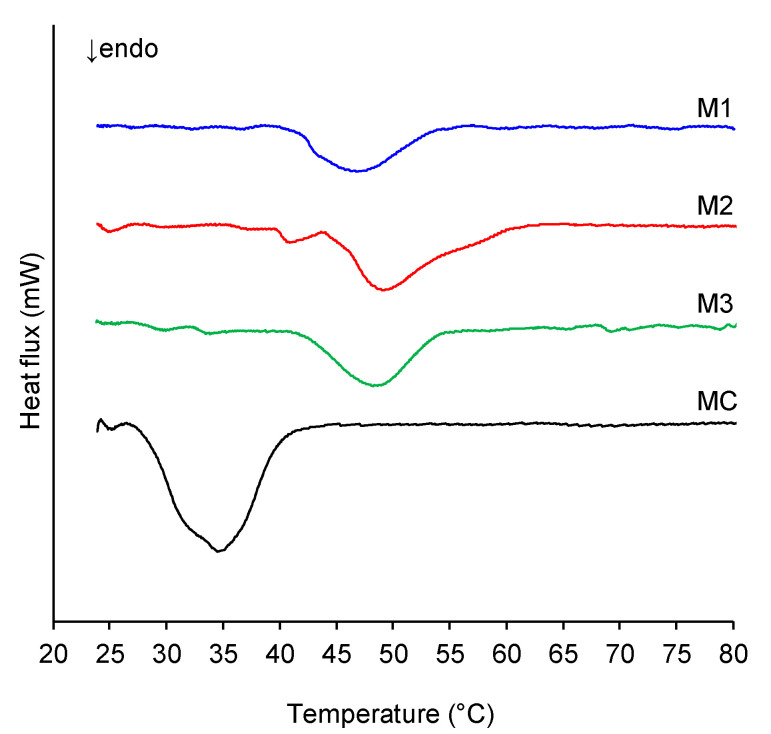
Melting profile (DSC) of the margarine formulations expressed in milliwatts (mW) as a function of temperature.

The melting profiles of the margarine formulations (M1, M2, M3) and the commercial margarine (MC) are presented in Figure 1. The commercial margarine exhibited a lower melting temperature range, with a peak melting temperature (T_p_) of 34.46 °C, compared to all oleogel-based margarines. Initially, both commercial and oleogel margarines had similar T_p_ values, as also reported in previous studies [16,23]. However, the oleogel margarines presented a broader and higher melting range, approximately between 42 °C and 55 °C (Tp ranging from 46 °C to 49 °C). This elevated melting temperature is likely attributable to the melting point of beeswax, which acts as the primary structuring agent in the oleogel formulations [16,30,31].

The formulations containing oleogels exhibited onset melting temperatures (T_onset_) between 42 and 45 °C, with maximum melting peak temperatures of 46.69 °C, 48.26 °C, and 49.21 °C for M1, M3, and M2, respectively. The higher T_p_ values for M3 and M2 correspond to their greater beeswax content. M1, which had the highest proportion of olive oil and the lowest wax concentration among the oleogel-based formulations, showed a comparatively lower melting range. A notable difference was also observed in the enthalpy values: the commercial margarine had an enthalpy of 5.20 mW, whereas the oleogel margarines ranged from 1.9 to 2.8 mW. This reduced enthalpy in the oleogel samples is commonly attributed to their higher unsaturated oil content [23], with M1, containing the most olive oil, presenting the lowest enthalpy (1.9 mW). From a practical perspective, oleogel-based margarines demonstrate greater stability at room temperature, enabling them to remain unrefrigerated for longer periods without melting or losing creaminess—qualities that are highly desirable for consumers in everyday use [16,23].

### 2.3. Color

**Figure 2:**
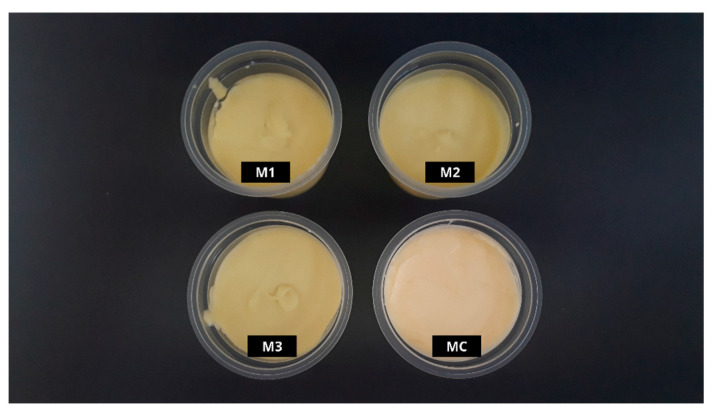
Visual appearance of the evaluated margarine formulations.

Visual attributes are among the primary and most noticeable characteristics perceived in a product [32]. Consequently, color is a crucial factor influencing consumer acceptance of new products. Figure 2 illustrates the visual appearance of the margarine formulations evaluated in this study.

**Figure 3:**
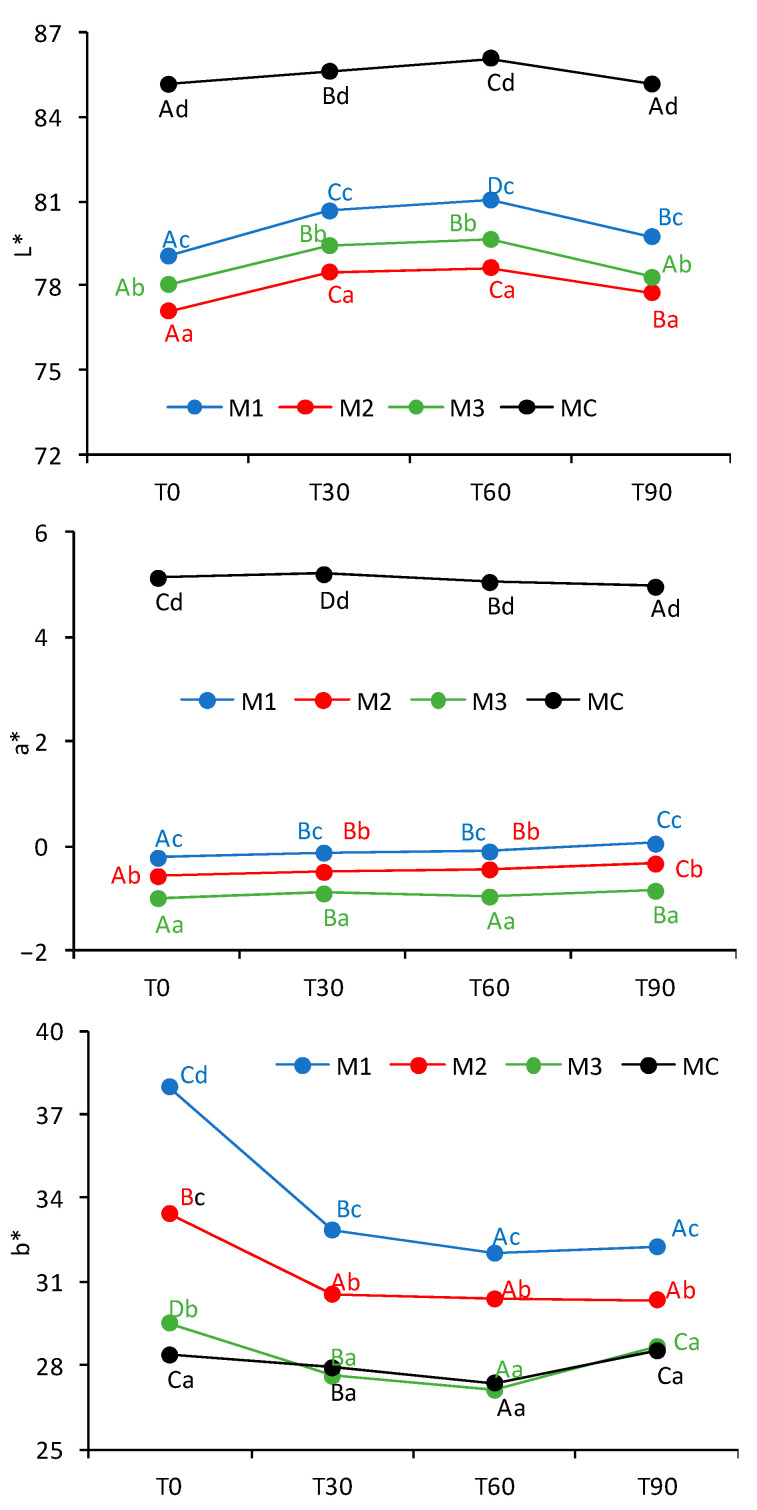
Color parameters of the margarines over time: L* (lightness), a* (red-green), and b* (yellow-blue), shown from top to bottom. Uppercase letters indicate statistical differences within each formulation over time, while lowercase letters denote comparisons between formulations at each time point.

Initially, formulations M1, M2, and M3 exhibited similar color characteristics, which appeared slightly lighter than those of the commercial margarine—an observation confirmed by instrumental analysis. Figure 3 presents the results for lightness (L*), the green-red axis (a*), and the blue-yellow axis (b*), highlighting the differences among all formulations as well as their changes over time.

The lightness parameter (L*) ranged from 77 to 86 across all formulations. Significant differences in L* values were observed among the samples at each time point. The ascending order of lightness was M2 < M3 < M1 < MC, suggesting that beeswax had a substantial influence on luminosity. All formulations exhibited a significant increase in lightness after 30 and 60 days of storage, followed by a decrease at 90 days, indicating a gradual loss of lightness during the shelf life of both the commercial and oleogel-based margarines.

Similarly, the a* values varied significantly among formulations at all time points. The oleogel-based margarines had negative a* values, indicating a shift toward green tones, whereas the commercial margarine exhibited positive a* values, reflecting a redder hue. This difference is likely attributable to the presence of extra virgin olive oil in the oleogel formulations [9,16].

Regarding the b* coordinate, although significant differences were observed among samples, all values were positive, ranging from 27 to 37, indicating a consistent yellow hue—characteristic of margarine products. Among the oleogel formulations, M2 showed the least variation in b* values over time, while M3’s b* values closely matched those of the commercial sample throughout the study.

Comparable results were reported in previous studies for the b* coordinate; however, the a* values differed, as all their formulations exhibited positive a* values [23]. The variations in a* among the oleogel formulations in the present study may be due to experimental variability or interactions with other formulation components whose concentrations differed. Despite minor shifts in color parameters, the product remained within acceptable visual quality standards [23].

### 2.4. Thermal Cyclization

**Figure 4:**
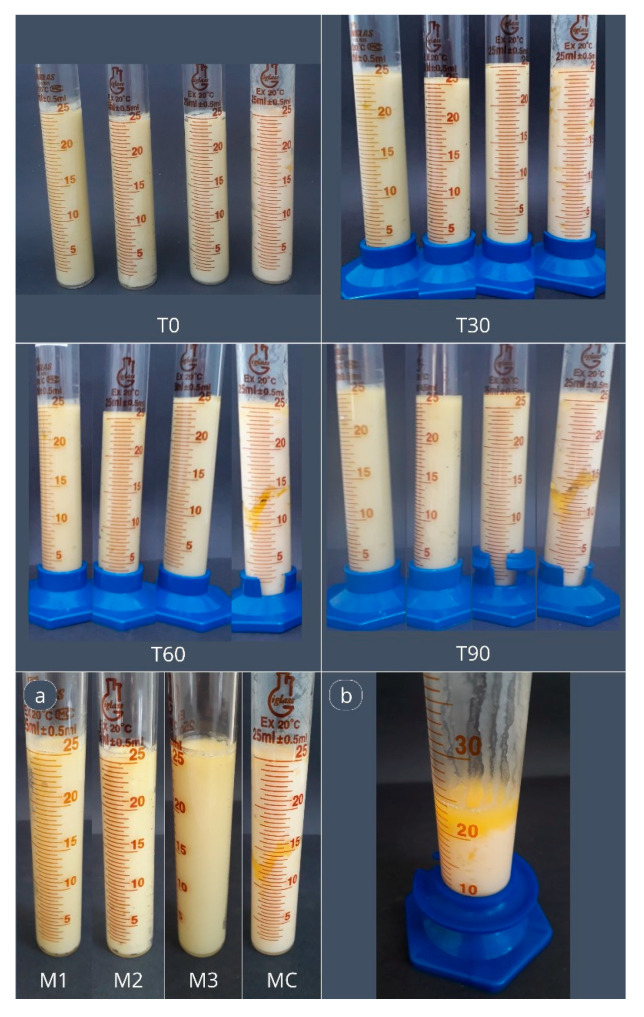
Margarine formulations (from left to right: M1, M2, M3, MC) stored in test tubes at times T0, T30, T60, and T90, and after the final cyclization stage: (**a**) Formulations M1, M2, M3, and MC at the end of the storage period (T90). (**b**) Oil exudation observed in sample MC at T90.

The formulations were subjected to thermal cycling to evaluate their stability under elevated temperatures and fluctuating storage conditions. Figure 4 presents the applied storage conditions and depicts the appearance of the formulations at the conclusion of the experiment.

Based on the presented images, the standard commercial sample exhibited the lowest stability, as evidenced by substantial oil exudation in the central and upper regions of the product. This exudation progressively increased, reaching approximately 4 milliliters (mL) by the conclusion of the experiment (Figure 4). Furthermore, the commercial margarine was unable to regain its structure upon returning to 5 °C, regardless of the duration of exposure. In contrast, the oleogel-based margarines did not exhibit any oil or water exudation. Overall, a very slight onset of exudation was observed after 24 h at 35 °C; however, following the final stage of thermal cycling (72 h at 5 °C), the samples reverted to their initial condition. These findings are consistent with the melting profiles of the margarines and reinforce the observation that the commercial sample was more susceptible to temperature fluctuations, particularly during extended storage periods.

The commercial margarine sample also demonstrated lower resistance to temperature fluctuations, which led to oil exudation in several instances [23,33]. In some cases, emulsion breakdown was observed. This instability may be attributed to the absence of a robust crystalline network, as found in the oleogel-based formulations. The oleogels likely formed a strong crystal network capable of effectively entrapping oil through the action of the structuring agent and other formulation components, thereby preventing phase separation even under thermal stress. These findings, in combination with the melting profile analyses, suggest that margarines formulated with oleogels exhibited greater stability than the commercial margarine, making them less susceptible to temperature variations such as those associated with alternating storage between refrigeration and ambient conditions.

### 2.5. Microstructure

**Figure 5:**
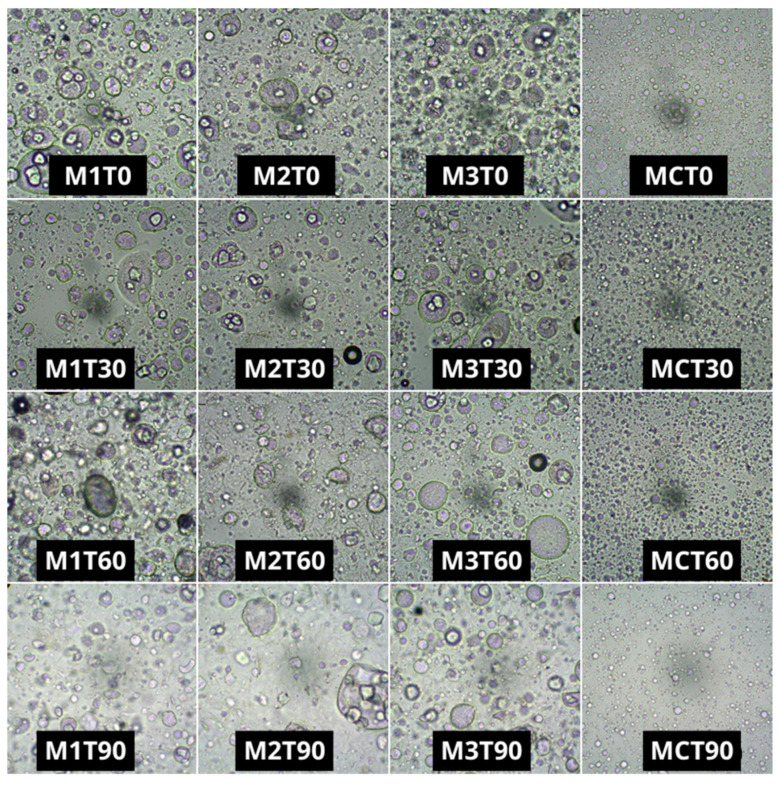
Microstructure of the margarine formulations observed over 90 days of storage.

The microstructure of the margarine samples was assessed both qualitatively and quantitatively by measuring the mean diameter of water droplets. Staining confirmed the formation of a water-in-oil (W/O) emulsion, with water droplets dispersed throughout the lipid phase. The microstructural characteristics of the samples are presented in Figure 5.

**Figure 6:**
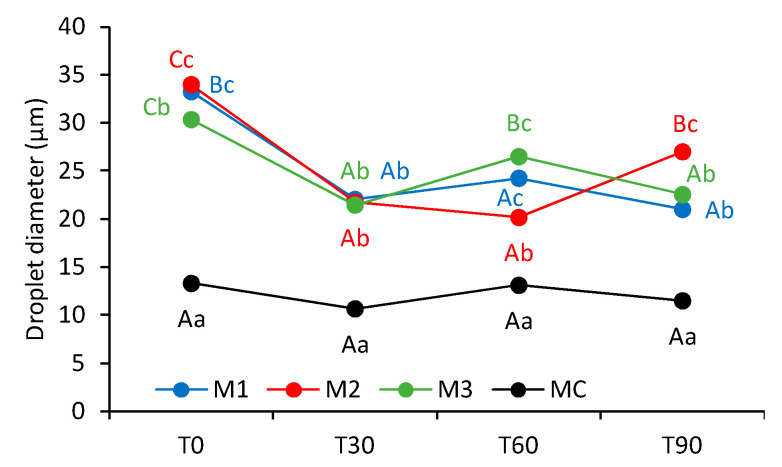
Average droplet diameter of the margarine formulations over time. Uppercase letters indicate statistical differences within each formulation over time, while lowercase letters compare different formulations at the same time point.

At the initial time point, a clear distinction was observed between the microstructure of the oleogel-based margarines and that of the commercial margarine. In all the formulations, a general trend was noted wherein the number of water droplets decreased while their size increased over time. However, the commercial margarine consistently displayed smaller droplets in higher numbers compared to the oleogel-based samples. These findings are supported by the data shown in Figure 6, which present the average droplet diameter (DD). Although variations in DM were observed throughout the storage period, these changes were not statistically significant (*p* > 0.05).

In general, the formulations M1, M2, and M3 had mean diameters between 20 and 34 µm, while the commercial margarine had an average of 12.16 µm, possibly due to the difference in the margarine production process. Nevertheless, the margarines formulated with oleogels remained stable, with similar textures and without oil exudation. Additionally, in M1, M2, and M3, the water droplets were more irregular, larger, and more dispersed in the visualized space. A disorganized aqueous phase characterized by large droplets has been previously reported in nonpolarized microstructure analyses [23]. This confirms, once again, a greater uniformity, including structurally, of the commercial sample, which probably did not negatively influence its stability over time, as seen in the thermal and oxidative analyses. These irregularities became more pronounced over time, with visually larger and more agglomerated droplets observed. Nevertheless, the mean droplet diameter did not change significantly over the storage period (Figure 6).

Although the oleogel-based formulations appeared less regularly structured and less homogeneous under microscopic observation, they were not less stable, contrary to what might be expected given the typical correlation between microstructure and macroscopic properties. In fact, as demonstrated in the thermal stability analyses, these formulations exhibited greater resistance to thermal fluctuations. This enhanced stability may be attributed to the synergistic effect of the CS-BW-CO combination. When used alone at similar concentrations, beeswax has been reported to produce margarines with structural instability [28]. Previous studies have shown that combining BW with a second wax can improve structural integrity; however, such combinations may also intensify a waxy mouthfeel. In contrast, the use of starch in combination with waxes may result in improved sensory properties and overall product acceptability [34].

### 2.6. Oxidation Evaluation

**Figure 7:**
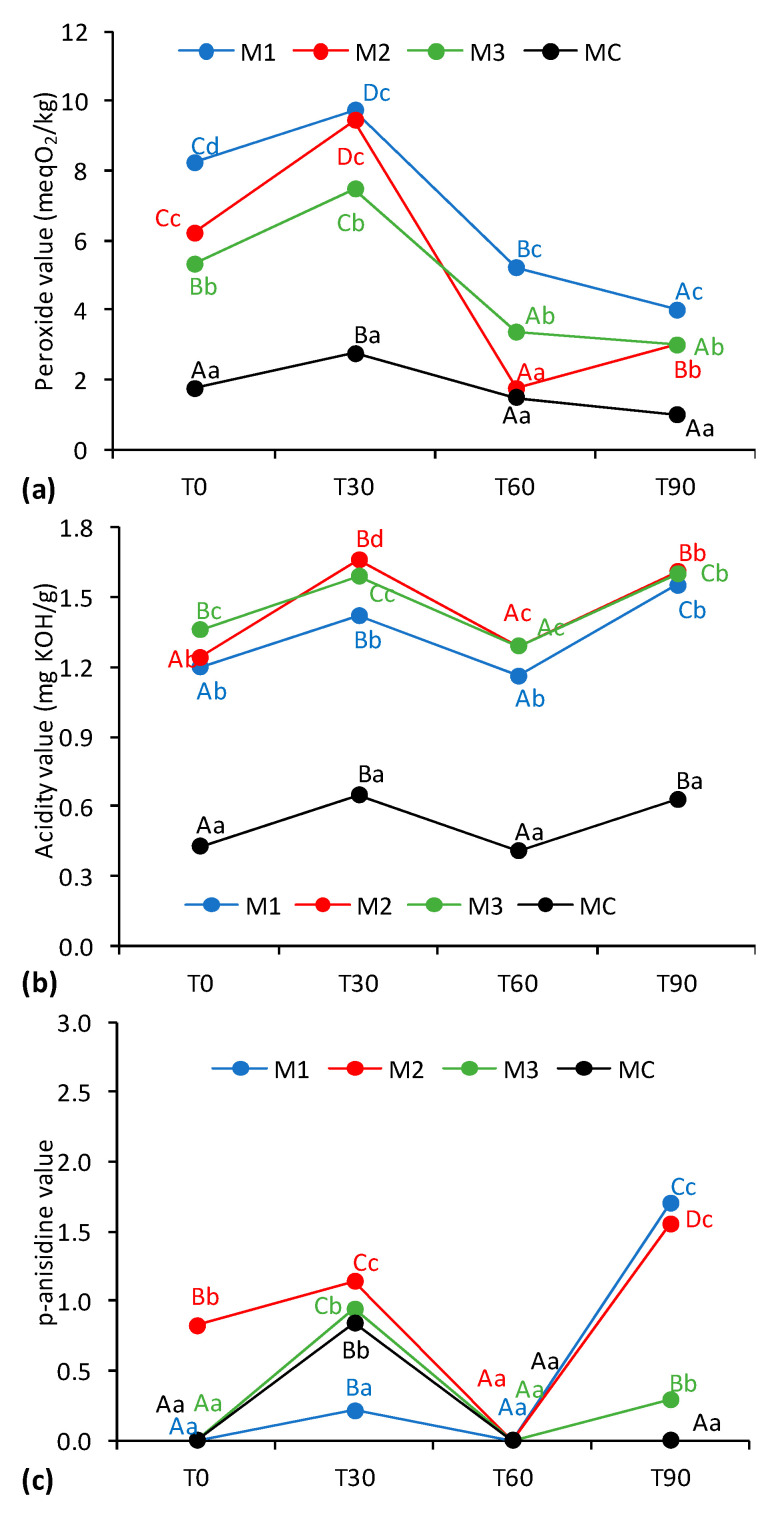
Lipid stability based on (**a**) peroxide value, (**b**) acidity value, and (**c**) p-anisidine value of the margarine formulations over time. Uppercase letters indicate statistical differences within each formulation across time points, and lowercase letters indicate differences between formulations at the same time point.

Foods with high lipid content are particularly susceptible to spoilage, primarily due to oxidative processes [35]. The peroxide value (PV) is a key indicator used to assess fat rancidity, reflecting the extent of primary lipid oxidation. In the food industry, oxidation and the resulting rancidity negatively impact product quality and compromise various functional properties [23]. The PV measurements for the margarine samples are presented in Figure 7a.

With the exception of the measurement at T60, the commercial margarine exhibited a significantly lower PV compared to the other samples, with an average PV of 1.74 mEq O_2_/kg. In contrast, the PV of the oleogel-based margarines ranged from 1.74 to approximately 9.73 mEq O_2_/kg. The higher PV observed in the oleogel margarines is likely attributable to the inclusion of extra virgin olive oil, for which a PV ≤ 20 mEq O_2_/kg is generally considered acceptable [36]. The initial PV (T0) was higher in samples with greater amounts of olive oil, a trend explained by the higher unsaturated fat content of olive oil relative to coconut oil or the commercial fat blend used in the commercial margarine.

A substantial increase in PV was observed during the first 30 days of storage, particularly in the oleogel-based formulations, followed by a subsequent decline. This decrease may be attributed either to the activity of antioxidants halting the propagation of peroxides or to the progression of oxidation into secondary stages. In the commercial margarine, multiple antioxidants and additional preservatives are included, whereas the formulations developed in this study contained only a single antioxidant and no industrial preservatives. In a previous study [37], significantly lower PVs and no notable changes in oleogel samples during storage were reported—findings attributed to the natural antioxidant compounds present in extra virgin olive oil.

Margarine contains water, which can influence oxidative stability during storage [37]. When peroxide value analysis was conducted on samples subjected to the thermal cycling process, the resulting values were slightly elevated but did not exceed the maximum recorded in Figure 7a, which was approximately 5.77 mEq O_2_/kg. Nevertheless, as previously discussed, although the oleogel-based margarines exhibited higher PVs than the commercial sample, the values remained within acceptable limits, particularly considering the inclusion of extra virgin olive oil in the formulations.

Figure 7b presents the acidity values measured throughout the storage period. The commercial margarine consistently showed significantly lower acidity compared to all other formulations, with an average value of 0.53 mg KOH/g. In contrast, the oleogel-based margarines exhibited some variation in acidity over the 90-day storage period; however, these fluctuations were gradual and not statistically significant at specific time points. Notably, the acidity values of the oleogel margarines remained stable, ranging from 1.20 to 1.70 mg KOH/g, indicating good chemical stability and the absence of spoilage. Furthermore, the thermal cycling treatment did not significantly alter the acidity index, suggesting that this process preserved the chemical integrity of the samples. These results indicate that while the incorporation of oleogels may influence initial acidity, all formulations maintained acceptable acidity levels throughout storage.

Secondary oxidation products, resulting from lipid degradation, were assessed using the p-anisidine value, as shown in Figure 7c. The average p-anisidine values were low, ranging from 0 to 1.70, indicating minimal formation of secondary oxidation products during storage, especially considering that values below 10 are commonly regarded as acceptable. Similar to the PV trend, the p-anisidine values declined after day 30 (T30), with all formulations approaching a value of 0. This reduction may be attributed to antioxidant activity, preventing the formation of secondary oxidation compounds. However, these slight variations remain within a narrow range. Among the oleogel formulations, M3—containing the lowest percentage of olive oil—exhibited the smallest increase in p-anisidine value after T60. This suggests that the observed decrease in PV for formulations M1 and M2 during storage was likely due to the progression of lipid oxidation into secondary stages. In samples subjected to thermal cycling, a further decrease in the p-anisidine values of M1 and M2 was observed, while an increase occurred in M3 and MC.

## 3. Conclusions

The production of margarines using oleogels based on extra virgin olive oil, coconut oil, starch, and wax is a feasible approach, particularly due to the favorable appearance, thermal stability, and oxidative stability of the resulting products. The presence of bioactive compounds in extra virgin olive oil likely contributed to enhanced oxidative stability, as the oleogel-based formulations did not exhibit elevated oxidation values throughout the study. Additionally, the structuring agents may have played a role in protecting the lipid components of olive oil from oxidation during storage. As expected, attributes such as color were only minimally influenced by the ingredients used, which is unlikely to negatively affect consumer perception. The oleogel-based margarines met established identity and quality standards, making them a promising and appealing alternative for the food industry—especially in response to growing consumer demand for healthier fat sources.

## 4. Materials and Methods

### 4.1. Materials

Extra virgin olive oil was supplied by Irarema Farm (Poços de Caldas, Minas Gerais, Brazil). The monoglyceride VEROL N-90 was provided by Lasenor (Potim, São Paulo, Brazil), and the butter flavoring was obtained from Doremus (Guarulhos, São Paulo, Brazil). The antioxidant butylhydroxytoluene (BHT) was acquired from Mix das Essências (Belo Horizonte, Minas Gerais, Brazil), while beeswax was purchased from Fenix Ceras (São Paulo, São Paulo, Brazil). Coconut oil, powdered milk, salt, corn starch, commercial margarine, and turmeric (used as a coloring agent) were procured from a local market in Lavras, Minas Gerais, Brazil. Methylene blue and Sudan III were used as dyes for microstructural analysis. Reagents employed in the oxidation analyses included sodium thiosulfate, starch, potassium hydroxide (KOH), and phenolphthalein, all obtained from Êxodo Científica. All ingredients were stored according to the manufacturers’ recommendations until use in margarine preparation.

### 4.2. Formulation of the Oleogel-Based Margarines

The final margarine formulations were prepared according to the compositions outlined in Table 4. Each formulation consisted of 80% lipid phase and 20% aqueous phase. A simplified production method, adapted for the incorporation of oleogels, was employed based on the procedure described by da Silva [23].

#### 4.2.1. Experimental Design

The concentrations of the structural components were initially defined through preliminary testing, followed by a Central Composite Rotational Design (CCRD) to establish the minimum and maximum concentration limits required to obtain spreadable margarines. The CCRD was employed as an optimization tool. Seventeen margarine formulations (Table 1) were prepared following the procedure detailed in Section 4.2.2. These formulations, along with a commercial reference sample, were stored under refrigeration and characterized using a texture analyzer (TA-XT.plus, Stable Microsystems, England). The optimized variables included the contents of corn starch (CS), beeswax (BW), extra virgin olive oil (OO), and coconut oil (CO), while the remaining formulation components were kept constant.

The optimization objective was to achieve a texture most similar to that of commercial margarine. This target was quantified using a desirability function with the criterion “Nominal-The-Best” [25]. Firmness, adhesiveness, and consistency values were transformed into desirability scores ranging from 0 to 1, with a value of 1 indicating the closest match to the desired texture. All texture measurements were performed in duplicate. The CCRD data were analyzed using Chemoface software version 1.71 [38], applying a quadratic model based on pure error estimation.

#### 4.2.2. Margarine Preparation

All ingredients were weighed according to the formulations presented in Table 4. The aqueous phase ingredients were manually mixed and heated to 60 °C in a water bath (HH-S3 Warmnest, Brazil). Simultaneously, the lipid-phase components were pre-mixed and heated to 80 °C in a separate water bath for approximately 10 min until fully solubilized. The two phases were then combined and homogenized at 1400 revolutions per minute (rpm) using an overhead agitator (713D, Fisatom, Brazil) for 20 min to ensure complete emulsification.

Following homogenization, the emulsions underwent a rapid dynamic cooling process (~10 °C/min) to room temperature (25 ± 1 °C) using a water bath maintained at 10 ± 1 °C, while being stirred with the overhead agitator at 200 rpm for 5 min. The resulting margarines were transferred into plastic containers, sealed with aluminum foil, appropriately labeled, and stored under refrigeration at 5 ± 1 °C until further analysis.

Samples were evaluated over a 90-day storage period at intervals of 0 (T0), 30 (T30), 60 (T60), and 90 (T90) days. All subsequent analyses were performed at each storage interval, with the exception of differential scanning calorimetry (DSC), which was conducted only at the start of the experiment.

### 4.3. Thermal Behavior

Differential Scanning Calorimetry (DSC) was performed to evaluate the melting behavior of the margarine samples. The analysis was conducted using a DSC-60A calorimeter equipped with a TA-60WS data acquisition system and an FC-60A furnace flow and atmosphere controller (Shimadzu, Japan). Approximately 5 mg of each margarine sample was weighed into a non-sealed aluminum pan. The samples were introduced into the DSC at room temperature (~25 °C), which was considered the onset temperature (T_onset_). The heating protocol involved a linear temperature increase from 25 °C to 90 °C at a rate of 2 °C/min. The melting peak temperature (T_p_) was recorded and used as the primary parameter for comparing the samples. All measurements were conducted under a nitrogen (N_2_) atmosphere to prevent oxidative degradation during the analysis.

### 4.4. Color Analysis

Color analysis of the margarine samples was performed using a spectrophotometer (CM-5, Konica Minolta, Japan). Samples were placed in 50 mL graduated glass beakers, and color measurements were conducted based on the CIELAB color space, recording the L*, a*, and b* values. The L* value represents lightness, ranging from 0 (black) to 100 (white). The a* coordinate indicates chromaticity along the green (negative) to red (positive) axis, while the b* coordinate reflects the blue (negative) to yellow (positive) axis. Measurements were carried out in triplicate using a D65 standard illuminant and a 10° observer angle.

### 4.5. Thermal Stability by Cyclization

The margarines were evaluated for emulsion stability—specifically, oil or water exudation—at various storage times and temperatures using a modified thermal cycling method adapted from [33]. Triplicate samples of approximately 25 g were placed in test tubes. To simulate temperature fluctuations, the samples were initially stored at 5 °C (T-34, Thelga, Brazil) for 48 h to ensure complete crystallization. They were then subjected to a temperature of 35 °C for 24 h. Following this period, the samples were returned to 5 °C for an additional 72 h. Visual inspections and, when possible, quantitative measurements of oil or water exudation were performed after each stage of the thermal cycle.

### 4.6. Microstructure Analysis

The microstructure of the margarine samples was analyzed using an optical microscope (BA210E, Motic, Spain) equipped with a 40× objective lens. To confirm the formation of an emulsion, a dual-staining technique was employed: methylene blue was used to stain the aqueous phase, and Sudan III was used to stain the lipid phase. These dyes were applied to the samples and examined microscopically to verify the presence of both phases, thereby confirming the formation of a water-in-oil (W/O) emulsion.

Following confirmation, the microstructure was further evaluated in the unstained, natural state. Small droplets were collected from various regions of each sample and placed onto glass slides, which were then sealed with coverslips. These slides were analyzed both qualitatively and quantitatively to assess the diameter of dispersed water droplets over time. For each sample, three slides were prepared, and quantitative measurements were conducted using ImageJ software (version 1.53, National Institutes of Health, Bethesda, MD, USA).

### 4.7. Lipid Stability and Oxidation

To evaluate lipid stability, margarine samples were preheated in an oven (MD1.1, Medicate, Brazil) at approximately 40 °C. Prior to analysis, the samples were filtered through filter paper to separate the lipid and aqueous phases; only the lipid phase was subjected to further analysis.

#### 4.7.1. Peroxide Index

The peroxide index (PI) was determined in triplicate using iodometric titration. Sodium thiosulfate was used as the titrant and starch as the indicator, following the AOCS official method Cd 8-53 [39].

#### 4.7.2. Acidity Index

The acidity index (AI) was measured in triplicate through acid–base titration, using potassium hydroxide (KOH) as the titrant and phenolphthalein as the indicator, in accordance with AOCS method Cd 3d-63 [39].

#### 4.7.3. Anisidine Value

The p-anisidine value was determined in triplicate based on a modified version of the AOCS method Cd 18-90 [40]. Approximately 1 g of each sample was diluted to a final volume of 10 mL in a volumetric flask prior to analysis.

### 4.8. Statistical Analysis

For statistical analysis, analysis of variance (ANOVA) was performed, followed by the Scott–Knott test. Both analyses were conducted using Sisvar version 5.6 [41], with a level of significance of 5%. In the figures, uppercase letters indicate statistical differences within each formulation over time, while lowercase letters represent differences among formulations at each specific time point.

## Figures and Tables

**Table 1:** Experimental design matrix used to evaluate the effects of the oleogel components on texture desirability of the formulated margarines. Experimental values are shown in parentheses.

Formulation	CS (g/100 g)	BW (g/100 g)	OO/CO (g/100 g)	Average Desirability
1	−1.00 (1.80)	−1.00 (2.80)	−1.00 (36.10/39.30)	0.00
2	−1.00 (1.80)	−1.00 (2.80)	1.00 (53.90/21.50)	0.85
3	−1.00 (1.80)	1.00 (5.20)	−1.00 (36.10/36.90)	0.40
4	−1.00 (1.80)	1.00 (5.20)	1.00 (53.90/19.10)	0.82
5	1.00 (4.20)	−1.00 (2.80)	−1.00 (36.10/36.90)	0.46
6	1.00 (4.20)	−1.00 (2.80)	1.00 (53.90/19.10)	0.36
7	1.00 (4.20)	1.00 (5.20)	−1.00 (36.10/45.50)	0.53
8	1.00 (4.20)	1.00 (5.20)	1.00 (53.90/16.70)	0.46
9	−1.68 (0.98)	0.00 (4.00)	0.00 (45.00/30.02)	0.87
10	1.68 (5.02)	0.00 (4.00)	0.00 (45.00/25.98)	0.76
11	0.00 (3.00)	−1.68 (1.98)	0.00 (45.00/30.02)	0.65
12	0.00 (3.00)	1.68 (6.02)	0.00 (45.00/25.98)	0.81
13	0.00 (3.00)	0.00 (4.00)	−1.68 (30.03/42.97)	0.52
14	0.00 (3.00)	0.00 (4.00)	1.68 (59.97/13.03)	0.47
15	0.00 (3.00)	0.00 (4.00)	0.00 (45.00/28.00)	0.20
16	0.00 (3.00)	0.00 (4.00)	0.00 (45.00/28.00)	0.10
17	0.00 (3.00)	0.00 (4.00)	0.00 (45.00/28.00)	0.00

CS: corn starch, BW: beeswax, OO: olive oil, CO: coconut oil.

**Table 2:** Regression coefficients of the quadratic model used to evaluate the effects of the oleogel components on texture desirability of the formulated margarines.

Coefficient	Value	Error	t	*p*
b_0_	0.12	0.03	4.22	0.000464
CS	−0.03	0.01	−2.54	0.019896
BW	0.06	0.01	4.55	2.19 × 10^−4^
OO/CO	0.07	0.01	5.70	1.70 × 10^−5^
CS × BW	−0.03	0.02	−1.49	0.152451
CS × OO/CO	−0.18	0.02	−10.68	1.81 × 10^−9^
BW × OO/CO	−0.05	0.02	−3.02	7.04 × 10^−3^
CS^2^	0.20	0.01	14.16	1.51 × 10^−11^
BW^2^	0.17	0.01	12.07	2.35 × 10^−10^
OO/CO^2^	0.09	0.01	6.30	4.79 × 10^−6^

CS: corn starch, BW: beeswax, OO: olive oil, CO: coconut oil.

**Table 3:** ANOVA for the quadratic model used to evaluate the effects of the oleogel components on texture desirability of the formulated margarines.

	GL	SQ	QM	F	*p*-Value
Regression	2.04	8	0.26	9.90	4.23 × 10^−6^
Residue	0.64	25	0.03		
Lack of fit	0.56	6	0.09	20.58	2.27 × 10^−7^
Pure error	0.09	19	0.00		
Total	2.69	33			
R^2^	0.76				
R^2^ adjusted	0.68				

**Table 4:** Formulation of the oleogel-based margarine used in the study.

Ingredients	g/100 g
**Aqueous phase 20%:**	
Water *	16.2
Salt *	2.0
Powdered milk *	1.8
**Lipid phase 80%:**	
Extra virgin olive oil	45–60
Coconut oil	40–55
Beeswax	4–5.2
Corn starch	1–1.8
Monoacylglycerol *	0.2
Turmeric *	0.03
Butter scent *	0.04
Antioxidant *	0.03

* fixed amount.

## Data Availability

The original contributions presented in this study are included in the article. Further inquiries can be directed to the corresponding author.
